# The association between active and passive tobacco smoke exposures and epilepsy in United States participants of the National Health and Nutrition Examination Survey (2013–2018)

**DOI:** 10.3389/fneur.2025.1502894

**Published:** 2025-05-09

**Authors:** Tingting Song, Chunlan Jia, Qi Wang, Jie Mu

**Affiliations:** ^1^Department of Neurology, West China Xiamen Hospital, Sichuan University, Xiamen, China; ^2^Center for Neurological Function Test and Neuromodulation, West China Xiamen Hospital, Sichuan University, Xiamen, China; ^3^Department of Neurology, West China Hospital, Sichuan University, Chengdu, China

**Keywords:** epilepsy, seizure, tobacco smoke exposures, cotinine, nicotine, NHANES

## Abstract

**Background:**

Epilepsy is a common chronic neurological disease, and identifying modifiable risk factors for epilepsy and seizure is extremely important. Currently, the relationship between tobacco exposure and epilepsy or seizure is controversy.

**Objective:**

The objective of this study is to test the relationship between tobacco smoke exposures and epilepsy in United States (US) participants of the National Health and Nutrition Examination Survey (NHANES).

**Methods:**

This is a cross-sectional study using data from NHANES 2013–2018. We included all participants in these cycles and excluded those with missing variables. Weighted logistic regression models, weighted sensitivity analysis and weighted subgroup analysis were conducted to estimate the association between active and passive tobacco smoke exposures and epilepsy.

**Results:**

We included 15,277 participants in NHANES, of whom 131 reported with epilepsy [taking at least one antiseizure medication (ASM) for epilepsy and recurrent seizures]. The weighted mean age of individuals is 42.35 years, 49.08% (95% confidence interval [CI] 48.03–50.12) were male, 64.56% (95%CI 63.70–65.41) were Non-Hispanic White, and 59.95% (95%CI 58.98–60.92) were private insurance. The weighted prevalence of epilepsy was 0.82% (95%CI 0.60–1.11) and 0.60% (95%CI 0.42–0.86) in those with and without tobacco smoke exposures, respectively. After adjusting for covariates, active and passive tobacco smoke exposure was not associated with epilepsy [weighted adjusted odd ratio (OR) 1.16, 95% CI 0.68–1.98, *p*-value = 0.576] and the results remained in multiple sensitivity analyses. However, we found that tobacco exposure was a protective factor for epilepsy in those aged 40–50 (OR 0.23, 95%CI 0.10–0.53, *p*-value < 0.001).

**Conclusion:**

In summary, tobacco exposure was not associated with epilepsy in the US population and this result remained after adjusting for confounding factors, and the sensitivity analysis was robust. However, in stratified analysis, tobacco exposure was a protective factor for epilepsy patients aged 40–50.

## 1 Introduction

Epilepsy is one of the most common chronic neurological disorders, affecting approximately 70 million persons all around the world and posing significant challenges and burden to public health and quality of life (1). According to the global burden of epilepsy in 2016, epilepsy accounted for 13.5 million disability-adjusted life-years (DALYs) and was responsible for 0.56% of total DALYs globally (2, 3). The prevalence of epilepsy is higher in low-income countries than in high-income countries and males demonstrating a marginally elevated risk relative to females (1, 4). The incidence has a bimodal distribution, with two distinct peaks: in infants under 1 year of age and in people aged 50 years and older (1). Within the older adult population (>50 years), the incidence rate increases with increasing age, and the incidence rate of people over 70 years old is the highest (1). Epilepsy is complex and multifactorial, with both genetic and environmental factors playing crucial roles. The risk factors vary among different age groups. Brain developmental abnormalities typically occur in those with onset before adulthood, while epilepsy related to head trauma, infection, and tumors can occur at any age and cerebrovascular disease usually occur in older people (1).

The smoke generated during smoking consists of a complex mixture of compounds, with different effects on human health, such as nicotine, tar, carbon monoxide, and polycyclic aromatic hydrocarbons. Tobacco exposure has negative effect to some diseases such as stroke, coronary heart disease, and lung cancer, while in others such as inflammatory bowel diseases, it has positive effect (5–8).

Tobacco smoke exposure is complex in epilepsy because some studies considered proconvulsant effects while others suggested anticonvulsant effects (9, 10). A prospective study, using data from the Nurses’ Health Study II, included 116,363 women aged 25–42 years. They found that after adjusting for stroke and other factors, current cigarette smoking was associated with increased risk of seizure (RR 2.60, 95% CI 1.53–4.42) and past smoking was associated with increased risk of epilepsy (RR 1.46, 95% CI 1.01–2.12) compared with never smoking (10). A meta-analysis of Mendelian randomization (MR) studies assessing the causal association of smoking in a variety of diseases and a two-sample MR study of risk factors for epilepsy identified that genetic liability to smoking was associated with increased risk of epilepsy (11, 12). For individuals in older age, the Framingham Heart Study showed that smoking was not associated with an increasing risk of epilepsy, but the Atherosclerosis Risk in Communities (ARIC) study indicated that smoking increased the risk of late-onset epilepsy (13, 14). A small sample study found that smokers with epilepsy were four times more likely to experience seizures than non-smokers with epilepsy, and the risk of refractory epilepsy did not increase (15). Furthermore, smoking and nicotine have been known to decrease the serum levels and anticonvulsive effect of lamotrigine (16–18). However, a retrospective study on Chinese males with epilepsy found a beneficial effect of smoking on seizure control (9).

In recent years, the prevalence of both active and passive smoking has been steadily increasing, particularly among individuals with poorer overall health (19–21). This trend highlights the need to pay closer attention to the impact of tobacco exposure on these vulnerable populations. The prevalence of smoking among people with epilepsy ranges from 20 to 48%, with most studies indicating that this rate is higher compared to those without epilepsy (22–29). However, the impact of tobacco exposure on epilepsy remains controversial. This study aimed to investigate the relationship between tobacco smoke exposure and epilepsy.

## 2 Materials and methods

### 2.1 Data sources and study population

**Figure 1:**
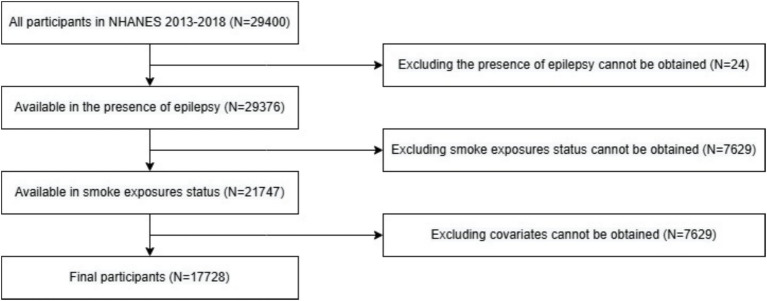
Flow chart.

This is a retrospective cross-sectional study using data from the National Health and Nutrition Examination Survey (NHANES) 2013–2018. The NHANES, a nationally representative survey, is conducted by the National Center for Health Statistics (NCHS) of Centers for Disease Control and Prevention (CDC) for assessing the physical and nutritional status of US population. We included all participants from 2013–2014, 2015–2016, 2017–2018 cycles of NHANES across different age groups (*N* = 29,400) and excluded those epilepsy status, tobacco exposure status or covariates was unavailable (*N* = 24, 7,629 and 4,019, respectively) in this analysis. Ultimately, the study included 17,728 participants (Figure 1). The datasets used in this analysis can be obtained from the NHANES official website^1^. Moreover, all participants in NHANES had signed informed consent forms.

### 2.2 Definition of tobacco smoke exposures

In our study, tobacco exposures were divided into active and passive tobacco exposures. Participants who answered yes to the question “During the past 5 days, including today, did you smoke cigarettes, pipes, cigars, little cigars or cigarillos, water pipes, hookahs, or e-cigarettes?” or the concentrations of cotinine in serum greater than 10 μg/L were identified as having active tobacco exposures. Passive tobacco exposures were defined as those who did not smoke, had at least one household smoker, or had the concentrations of cotinine in serum greater than 0.05 μg/L. Cotinine is one of the main metabolites of nicotine and the half-life of cotinine is longer than nicotine (15–20 h vs. 0.5–3 h). Therefore, the concentrations of cotinine in body fluids (such as serum, urine, and saliva) can be used as biomarkers for active smoking or secondhand smoke exposures.

Tobacco exposure was classified as active or passive. Due to the lack of detailed information in NHANES, factors such as smoking frequency, duration, and intensity were not included in the analysis. We acknowledge this as a limitation, and future studies should incorporate these aspects to better understand their impact on epilepsy risk.

### 2.3 Definition of epilepsy

All participants in the NHANES were required to respond to the following question: “In the past 30 days, have you used or taken medication for which a prescription is needed? Do not include prescription vitamins or minerals you may have already told me about.” Participants were then asked to provide the complete name and main reasons of each prescription medication they used. In our analysis, we defined participants as patients with epilepsy if they responded taking at least one prescription medication for the reason of “epilepsy and recurrent seizures” (ICD-10-CM codes: G40). We observed that some medications were reported for “epilepsy and recurrent seizures” but were not standard anti-seizure medications (ASMs). Therefore, we did not take such medications into consideration (Supplementary Table 1). The definition of epilepsy is according to previous studies (30, 31).

### 2.4 Other covariates

Other covariates included in our study were age, sex, race-ethnicity, BMI, family poverty-income ratio, insurance status, the concentrations of cotinine and the survey cycle. The age of participants is recorded by year, and individuals 80 or over 80 are recorded as 80 years of age. The race-ethnicity was categorized as Mexican American, Other Hispanic, Non-Hispanic White, Non-Hispanic Black, and Other Race. Body Mass Index (BMI) was regarded as a continuous variable. We used family poverty-income ratio to represent the economic situation. It was calculated as the ratio of family (or individual) income to the poverty guidelines specific to the survey year. We obtained the insurance information from the Health Insurance questionnaire. Insurance status was classified into the following: without any health insurance, covered by private insurance, and covered by public insurance only. Public insurance includes Medicare, Medicaid, Children’s Health Insurance Program (CHIP), Indian Health Service, military health plan (Tricare/VA/Champ-VA), state-sponsored health plan, and other government insurance.

### 2.5 Statistical analysis

All analyses used sampling weights to indicate complex sampling designs according to the data analysis tutorials of NHANES. We excluded participants with missing variables from the analysis, which could potentially introduce selection bias. While this approach is a common practice to ensure data consistency and reliability, we acknowledge that it may limit the generalizability of our findings. The exclusion of missing data could introduce some bias, especially if the missing data are not missing at random. In future studies, more advanced statistical methods, such as multiple imputations or propensity score matching, could be employed to address missing data and better assess their impact on the results.

To describe continuous variables, the survey-weighted mean and SD were used if the data conforms to normal distribution, and median and interquartile ranges were reported otherwise. For categorical variables, we reported survey-weighted proportions and 95% confidence intervals (CIs). Weighted multiple logistic regression was used to estimate crude and adjusted odds ratios (ORs) and 95% CIs of the association between tobacco smoke exposures and epilepsy. In our analyses, we established three models. The first model was not adjusted, the second model was adjusted for age, gender, and race-ethnicity, and the third model was adjusted for age, sex, race-ethnicity, BMI, family poverty-income ratio category, insurance status, and survey cycle. However, we acknowledge the importance of verifying all assumptions, including checking for outliers and testing for independence of errors. Future studies should consider conducting diagnostic tests such as the Durbin-Watson test for autocorrelation and the use of leverage statistics for detecting influential outliers.

To further test our findings, we conduct two sensitivity analyses. Firstly, the limit of detection of cotinine is used as a cutoff for tobacco exposure (cotinine level <0.05 μg/L vs. cotinine level ≥0.05 μg/L). Secondly, the smoking status is categorized as none, passive tobacco smoke exposures, and active tobacco smoke exposures. Then weighted multiple logistic regression was performed. In sub-group analysis, we stratified by age group, sex, race-ethnicity, family poverty-income ratio category, and insurance status. Age subgroups were classified into ≤20 years old, 20 ~ 30 years old, 30 ~ 40 years old, 40 ~ 50 years old, 50 ~ 60 years old, and >60 years old. The variables used in the analysis are classified according to their measurement scales: continuous variables: Age, BMI, Cotinine; dichotomous variables: Epilepsy, Tobacco exposure; ordinal variables: Poverty-income ratio categories; nominal variables: Sex, Race/ethnicity; Interval/ratio variables: Insurance status; This classification is crucial for understanding the nature of the data and ensuring appropriate statistical methods are used. All data handling was conducted with RStudio software version 4.0.5 and statistical analyses were performed using StataMP version 18. *p*-values < 0.05 were deemed significant.

## 3 Results

### 3.1 Demographics characteristics of study participants

There were 29,400 individuals participated in NHANES survey between 2013 and 2018. Of these, 17,728 individuals were included in our analysis (as presented in Figure 1). Table 1 demonstrates the distribution of age, sex, race-ethnicity, poverty-income ratio, insurance status, BMI, serum cotinine, and epilepsy in those with and without tobacco smoke exposure. The weighted mean age of all individuals in our study is 42.35 years, and 47.03 years of those without tobacco smoke exposure and 37.65 years of those with exposure. Male participants are more likely to experience tobacco smoke exposure. The Non-Hispanic White group accounted for the most weighted proportion in total population, non-exposure group, and exposure group (64.56% [95%CI 63.70–65.41%] vs. 68.21% [95%CI 67.01%-69.38] vs. 60.89% [95%CI 59.67–62.10%]). More than 50% of study participants who have a poverty index more than 180% of the federal poverty guideline (66.11% [95%CI 65.23–66.98%] in total, 77.40% [95%CI 76.32–78.44%] in non-exposure group, 54.79% [95%CI 53.46–56.12%] in exposure group). No matter in which groups, study participants tended to have private insurance (59.95% [95%CI 58.98–60.92%] in total, 70.93% [95%CI 69.61–72.20%] in non-exposure group, 48.94% [95%CI 47.56–50.34%] in exposure group). The survey-weighted prevalence of participants who had epilepsy was 0.71% (95%CI 0.56–0.90%), and it is consistent with previous researches (1, 2). The proportions of epilepsy in participants with and without tobacco smoke exposures was 0.82% (95%CI 0.60–1.11%) and 0.60% (95%CI 0.42–0.86%), respectively.

**Table 1:** Demographic characteristics of participants in NHANES 2013–2018.

Characteristics	% (95%CI)			VIF
	Total (*N* = 17,728)	Tobacco smoke exposure		
		None (*N* = 7,919)	Any (*n* = 9,809)	
Age, mean (SD), y	42.35 (0.19)	47.03 (0.27)	37.65 (0.26)	1.33
Sex				1.00
Male	49.08 (48.03–50.12)	45.49 (43.93–47.04)	52.68 (51.30–54.05)	
Female	50.92 (49.88–51.97)	54.51 (52.96–56.07)	47.32 (45.95–48.70)	
Race/ethnicity				1.03
Mexican American	9.44 (9.03–09.86)	10.40 (9.79–11.04)	8.47 (7.94–9.04)	
Other Hispanic	5.94 (5.62–6.29)	6.33 (5.86–6.84)	5.55 (5.12–6.03)	
Non-Hispanic White	64.56 (63.70–65.41)	68.21 (67.01–69.38)	60.89 (59.67–62.10)	
Non-Hispanic Black	11.05 (10.65–11.46)	6.50 (6.07–6.96)	15.61 (14.94–16.31)	
Other Race	9.01 (8.55–9.49)	8.56 (7.95–9.21)	9.46 (8.79–10.18)	
Family Poverty-income ratio				1.03
FPIR < 1.3	23.89 (23.17–24.64)	14.39 (13.58–15.24)	33.43 (32.27–34.61)	
1.3 ≤ FPIR ≤ 1.8	9.99 (9.49–10.51)	8.22 (7.57–8.91)	11.77 (11.03–12.56)	
FPIR > 1.8	66.11 (65.23–66.98)	77.40 (76.32–78.44)	54.79 (53.46–56.12)	
Insurance				1.06
None	13.51 (12.89–14.15)	8.90 (8.18–9.67)	18.13 (17.17–19.15)	
Private	59.95 (58.98–60.92)	70.92 (69.61–72.20)	48.94 (47.56–50.34)	
Public (only)	26.54 (25.72–27.377)	20.18 (19.08–21.33)	32.92 (31.73–34.13)	
BMI, median (IQR), kg/㎡	26.70 (22.30–31.80)	27.50 (23.60–32.20)	26.00 (21.10–31.40)	1.32
Serum cotinine, median (IQR), μg/L	0.05 (0.01–1.51)	0.01 (0.011–0.20)	0.74 (0.09–126.00)	1.06
Epilepsy	0.71 (0.56–0.90)	0.60 (0.42–0.86)	0.82 (0.60–1.11)	—

To address potential multicollinearity issues, we checked the variance inflation factors (VIFs) for the covariates included in the regression models. None of the VIFs exceeded the commonly accepted threshold of 10, suggesting that multicollinearity is not a significant concern in our analysis.

### 3.2 Association between tobacco smoke exposures and epilepsy

We verified the association between tobacco exposure and epilepsy in Table 2. In our analysis, tobacco smoke exposures are not significantly associated with epilepsy. In the unadjusted analysis, the OR for epilepsy in participants with tobacco exposure is 1.37(95%CI 0.85–2.21, *p*-value = 0.196) compared to those without exposure. But the result is not statistically significant. When adjusting for age, sex, and race-ethnicity and adjusting for full covariates, the results remain not statistically significant (OR 1.57, 95%CI 0.95–2.59, *p*-value = 0.078 and OR 1.16, 95%CI 0.68–1.98, *p*-value = 0.576).

**Table 2:** Association between active and passive tobacco smoke exposures and epilepsy.

Exposure	Model 1		Model 2		Model 3	
	OR (95%CI)	*P*-value	OR (95%CI)	*P*-value	OR (95%CI)	*P*-value
Any tobacco exposure	1.37 (0.85–2.21)	0.196	1.57 (0.95–2.59)	0.078	1.16(0.68–1.98)	0.576
Sensitivity analyses						
Lower cutoff of cotinine (<0.015 μg/L)	1.01 (0.61–1.66)	0.972	1.12 (0.65–1.91)	0.688	0.80 (0.45–1.42)	0.454
Passive tobacco exposure	1.14 (0.61–2.12)	0.690	1.37 (0.72–2.59)	0.333	1.05 (0.53–2.06)	0.892
Active smoking	1.64 (0.97–2.77)	0.066	1.76 (1.01–3.08)	0.046	1.28 (0.72–2.25)	0.399

Model 1: unadjusted.

Model 2: adjusted for age, sex, and race-ethnicity.

Model 3: adjusted for age, sex, race-ethnicity, BMI, family poverty-income ratio category, insurance status, and survey cycle.

To further explore the relationship between tobacco smoke exposure and epilepsy, we conducted a sensitivity analysis. Firstly, we used cotinine cutoff (0.015 μg/L) as the boundary value to define tobacco smoke exposure status, and obtained similar results (OR 0.80, 95% CI 0.45–1.42). Furthermore, we performed another sensitivity analysis by dividing tobacco smoke exposure into none, active and passive. When adjusting for age, sex, and race-ethnicity, we observed the positive correlation between tobacco exposure and epilepsy (OR 1.76, 95% CI [1.01–3.08]). But after full adjustment, we found results similar to that of the main analyses (OR 1.28, 95%CI [0.72–2.25]). Thus, there were no statistically significant relationship between tobacco smoke exposure and epilepsy (Table 2). We tested the linearity assumption between continuous predictors and the logit of the outcome using the Box-Tidwell procedure, and found no significant violations.

### 3.3 Weighted subgroup analysis

**Figure 2:**
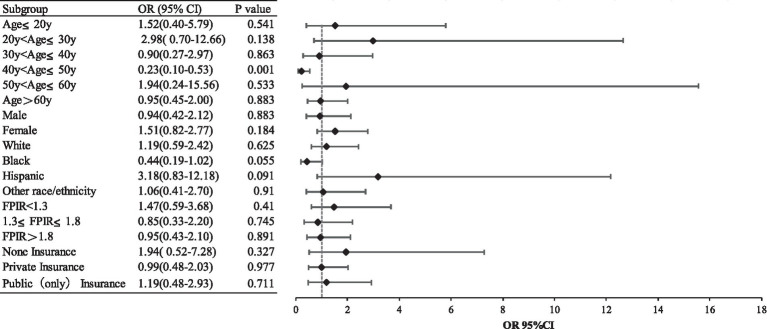
Subgroup analysis.

Moreover, we conducted weighted subgroup analyses to assess whether the association between tobacco smoke exposures and epilepsy was influenced by age group, sex, race-ethnicity, family poverty-income ratio category, and insurance status. We divided age into multiple groups and adjusted for all covariates, it was found that a significant negative correlation between tobacco smoke exposures and epilepsy was observed only in participants aged 40 ~ 50 years (OR 0.23, 95%CI 0.10–0.53). Conversely, the relationship was not statistically significant for those who were in other age groups or stratified by sex, race-ethnicity, family poverty-income ratio category, and insurance status. This finding was consistent with our main results (Figure 2).

## 4 Discussion

In this nationally representative study, 17,728 survey participants were included and the prevalence of epilepsy was 7‰, which is consistent with previous studies (4). We found no statistical association between tobacco exposure and epilepsy and the results persisted after adjusting for age, sex, race/ethnicity, body mass index category, poverty-income ratio category, insurance status, and survey year. In different subgroups of age, sex, and race/ethnicity, we only found a statistically significant association in the age-group of 40–50 years. The results were similar when using different definitions of tobacco exposure and in the group of active tobacco exposure or passive tobacco exposure.

In current literature, data on the relationship between tobacco smoke exposures and epilepsy are limited, and the association remains inconclusive. While it is widely accepted that tobacco exposure is a detrimental factor for individuals with epilepsy, our findings suggest that both active and passive tobacco smoke exposure may act as a protective factor for epilepsy in individuals aged 40–50 years. This result is similar to the research reported by Gao et al., but contrasts with other studies that identify tobacco exposure as a risk factor (9–14). These discrepancies may be attributed to differences in study populations, definitions of tobacco smoke exposure, and criteria for diagnosing epilepsy. First, many studies have been limited by small sample sizes, and none have utilized the NHANES database to explore the relationship between tobacco exposure and epilepsy. In contrast, our study employed data from the NHANES database (2013–2018) with weighted statistical analysis, providing national representativeness and a large sample size. Second, while previous studies typically relied on self-reported tobacco use, we assessed tobacco exposure through both self-reports and biomarkers. Additionally, we included participants exposed to both active and passive tobacco smoke. Finally, compared to earlier studies, we defined epilepsy based on the use of antiepileptic medications and the occurrence of recurrent seizures in the past 30 days.

The components of tobacco smoke are complex, and current studies have shown that some of these constituents, including nicotine, have anticonvulsant effects. Nicotine, a major alkaloid in tobacco, is rapidly absorbed into the bloodstream through the lungs and transported to the brain when tobacco products are smoked or used. In animal studies, low-dose nicotine has been shown to exert protective effects on seizure activity, suggesting a potential therapeutic role (32). The mechanisms through which nicotine influences epilepsy are multifaceted, and understanding these pathways is crucial for elucidating its clinical implications (33, 34). First, nicotine acts primarily through neuronal nicotinic acetylcholine receptors (nAChRs), which are ligand-gated ion channels widely distributed in both pre-and post-synaptic regions of the brain (33, 34). nAChRs modulate ion flux across cell membranes, influencing neuronal excitability and the release of various neurotransmitters (33, 34). This action impacts synaptic plasticity and other physiological and behavioral processes (33, 34). Dysregulation of nAChRs has been implicated in the pathophysiology of epilepsy, and nAChRs are considered potential therapeutic targets (33, 34). For instance, autosomal dominant nocturnal frontal lobe epilepsy (ADNFLE) is a rare form of epilepsy caused by mutations in genes encoding nAChRs (35). Given that nicotine is an agonist of nAChRs, its chronic exposure may alter receptor sensitivity, potentially offering therapeutic benefits for epilepsy linked to nAChR mutations (34, 36–38). Additionally, studies have demonstrated that nicotine and its metabolite, cotinine, can rapidly cross the blood–brain barrier and bind directly to MD2 proteins on microglial membranes (39, 40). This binding induces conformational changes in MD2, which may influence its function and reduce neuroinflammation caused by microglial activation (39, 40). Neuroinflammation is a key contributor to the onset and progression of epilepsy, and by modulating this pathway, nicotine may reduce seizure susceptibility and protect against long-term neurological damage (41). This anti-inflammatory effect presents a novel avenue for epilepsy treatment, although the dual effects of nicotine (i.e., both potential benefits and risks) must be carefully balanced in clinical practice. Furthermore, other tobacco smoke constituents, such as selenium, zinc, carbon dioxide, toluene, acetone, and nickel chloride, have also been shown to possess anticonvulsant properties, suggesting that nicotine may not be the only active component in tobacco with potential therapeutic effects (42–47). However, the complex nature of tobacco smoke and its varying effects on the brain necessitate further investigation to better understand its overall impact on epilepsy management. While nicotine has demonstrated potential anticonvulsant effects in certain studies, these findings should be interpreted with caution, given the potential limitations of sample size, study design, and other confounding factors. It is important to note that our results may be influenced by random variation or residual confounding, and additional studies with larger sample sizes and longitudinal designs are required to validate these findings. Further research is required to fully understand the biological mechanisms underlying these effects and their clinical implications for epilepsy treatment. This finding could lead to the development of novel therapies for drug-resistant epilepsy, although the risks associated with nicotine exposure, including addiction and adverse health effects, must be considered in clinical decision-making.

Our study has several limitations that should be noted. First, our definition of epilepsy was based on medication intake, which may not have captured all individuals with epilepsy, as some patients might have refused medication or discontinued it due to good control of their condition. However, multiple previous studies had used this definition, and there was evidence that the existence of ASM can greatly improve the detection of epilepsy in research datasets (30, 31, 48, 49). Second, the NHANES database does not provide detailed information on key characteristics of epilepsy, such as seizure frequency, duration, or classification. This limits our ability to fully assess the relationship between tobacco exposure and different subtypes of epilepsy. Third, there is a risk of residual confounding due to the lack of certain covariates, such as family history of epilepsy, history of head trauma, and past encephalitis or meningitis, which could influence the risk of epilepsy. These factors were not available in the NHANES dataset and could have affected the results. Fourth, due to the lack of detailed information in NHANES, factors such as smoking frequency, duration, and intensity were not included in the analysis. Finally, it is important to emphasize that this study is cross-sectional in nature and therefore cannot establish causality. The dataset does not provide information on the temporal relationship between tobacco exposure and the onset of epilepsy. Given the potential for random variation and the limitations in sample size, further studies are needed to validate our findings. There should be some better design, more confounders considered and longitudinal studies to be performed to investigate how tobacco smoke exposure affects the epilepsy network. In the future, extract from cigarette as adjunctive medication or nicotine therapy may help obtain better seizure control in patients.

## 5 Conclusion

In summary, tobacco exposure was not associated with epilepsy in the US population and this result remained after adjusting for confounding factors, and the sensitivity analysis was robust. However, in stratified analysis, tobacco exposure was a protective factor for epilepsy patients aged 40–50. More studies are need to investigate how smoking affects the epilepsy network in the brain, as well as a prospective study that considers more confounding factors to increase evidence of the association between tobacco exposure and epilepsy.

## Data Availability

The raw data supporting the conclusions of this article will be made available by the authors, without undue reservation.

## Footnotes

^1^ https://wwwn.cdc.gov/nchs/nhanes/Default.aspx
