# Supplementary material for: The association between active and passive tobacco smoke exposures and epilepsy in United States participants of the National Health and Nutrition Examination Survey (2013–2018)
Source: Front Neurol. 2025 May 9;16:1502894. doi: 10.3389/fneur.2025.1502894 (PMC12098089; doi:10.3389/fneur.2025.1502894)
Supplement: Supplementary file 1 [file Table_1.docx]

Supplementary Material

# Supplementary Table 1: We listed the medications reported for treating “epilepsy and recurrent seizures” in NHANES 2013-2018 (Some are ASMs, but others are non-ASMs).

| **ASMs** | **Non-ASMs** |
| --- | --- |
| Lamotrigine | Allopurinol |
| Phenobarbital | Hydrocodone |
| Carbamazepine | Venlafaxine |
| Clonazepam | Apixaban |
| Phenytoin | Tamsulosin |
| Gabapentin | Memantine |
| Levetiracetam | Losartan |
| Lorazepam | Buspirone |
| Lacosamide | Leucovorin |
| Clobazam | Carbidopa; Levodopa |
| Oxcarbazepine | Sertraline |
| Diazepam | Risperidone |
| Divalproex sodium |  |
| Topiramate |  |
| Valproic acid |  |
| Primidone |  |
| Zonisamide |  |
| Rufinamide |  |
| Pregabalin |  |
| Alprazolam |  |

**
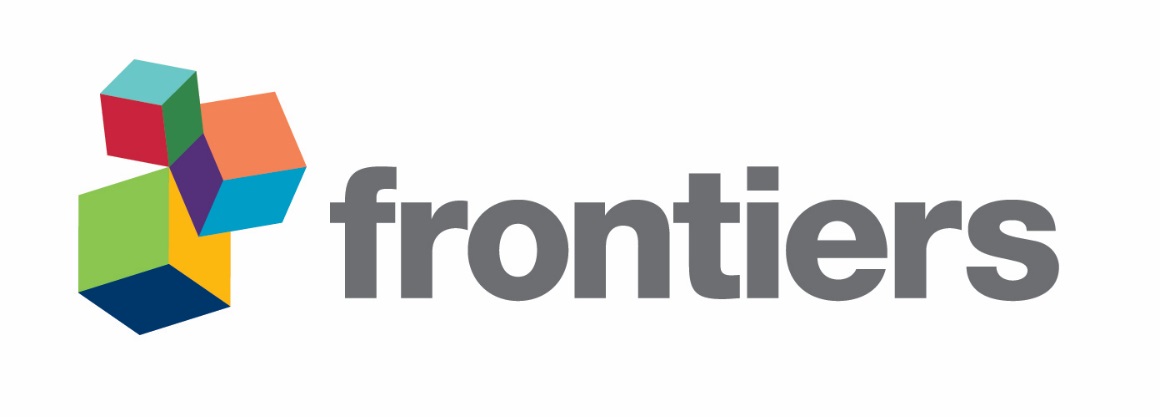
**
